# Supplementary material for: Sibling chimerism among microglia in marmosets
Source: eLife. 2026 Aug 14;13:RP93640. doi: 10.7554/eLife.93640 (PMC13476068; doi:10.7554/eLife.93640)
Supplement: Supplementary file 1 [file elife-93640-mdarchecklist1.docx]

**Materials Design Analysis Reporting (MDAR)**

**Checklist for Authors**

The [MDAR framework](https://osf.io/xfpn4/) establishes a minimum set of requirements in transparent reporting mainly applicable to studies in the life sciences.

*eLife* asks authors to **provide detailed information within their article** to facilitate the interpretation and replication of their work. Authors can also upload supporting materials to comply with relevant reporting guidelines for health-related research (see [EQUATOR Network](http://www.equator-network.org/%20)), life science research (see the [BioSharing Information Resource](http://biosharing.org/)), or animal research (see the [ARRIVE Guidelines](http://www.plosbiology.org/article/info:doi/10.1371/journal.pbio.1000412) and the [STRANGE Framework](https://doi.org/10.1038/d41586-020-01751-5); for details, see *eLife*’s [Journal Policies](https://reviewer.elifesciences.org/author-guide/journal-policies)). Where applicable, authors should refer to any relevant reporting standards materials in this form.

For all that apply, please note **where in the article** the information is provided. Please note that we also collect information about data availability and ethics in the submission form.

**Materials:**

| **Newly created materials** | **Indicate where provided: section/figure legend** | **N/A** |
| --- | --- | --- |
| The manuscript includes a dedicated "materials availability statement" providing transparent disclosure about availability of newly created materials including details on how materials can be accessed and describing any restrictions on access. |  | N/A |
|  |  |  |
| **Antibodies** | **Indicate where provided: section/figure legend** | **N/A** |
| For commercial reagents, provide supplier name, catalogue number and [RRID](https://scicrunch.org/resources), if available. |  | N/A |
|  |  |  |
| **DNA and RNA sequences** | **Indicate where provided: section/figure legend** | **N/A** |
| Short novel DNA or RNA including primers, probes: Sequences should be included or deposited in a public repository. |  | N/A |
|  |  |  |
| **Cell materials** | **Indicate where provided: section/figure legend** | **N/A** |
| Cell lines: Provide species information, strain. Provide accession number in repository OR supplier name, catalog number, clone number, OR RRID. |  | N/A |
| Primary cultures: Provide species, strain, sex of origin, genetic modification status. | Methods section (Whole Genome Sequencing; Donor-of-origin analysis): primary fibroblast cultures were derived from marmoset (Callithrix jacchus) tissue biopsies, used solely to obtain chimerism-free reference genotypes by whole-genome sequencing; no genetic modification. |  |
|  |  |  |
| **Experimental animals** | **Indicate where provided: section/figure legend** | **N/A** |
| Laboratory animals or Model organisms: Provide species, strain, sex, age, genetic modification status. Provide accession number in repository OR supplier name, catalog number, clone number, OR RRID. | Methods (Ethical compliance) and Table 1: species (Callithrix jacchus, common marmoset), sex, age and colony of origin (NEPRC, CLEA Japan, or a non-clinical contract research organization) are reported for each of the 11 marmosets studied; animals were obtained from established breeding colonies. |  |
| Animal observed in or captured from the field: Provide species, sex, and age where possible. |  | N/A |
|  |  |  |
| **Plants and microbes** | **Indicate where provided: section/figure legend** | **N/A** |
| Plants: provide species and strain, ecotype and cultivar where relevant, unique accession number if available, and source (including location for collected wild specimens). |  | N/A |
| Microbes: provide species and strain, unique accession number if available, and source. |  | N/A |
|  |  |  |
| **Human research participants** | **Indicate where provided: section/figure legend) or state if these demographics were not collected** | **N/A** |
| If collected and within the bounds of privacy constraints report on age, sex, gender and ethnicity for all study participants. |  | N/A |

**Design:**

| **Study protocol** | **Indicate where provided: section/figure legend** | **N/A** |
| --- | --- | --- |
| If the study protocol has been pre-registered, provide DOI. For clinical trials, provide the trial registration number OR cite DOI. |  | N/A |
|  |  |  |
| **Laboratory protocol** | **Indicate where provided: section/figure legend** | **N/A** |
| Provide DOI OR other citation details if detailed step-by-step protocols are available. | Methods. Nucleus Drop-seq library preparation and sequencing: step-by-step protocol available at protocols.io, DOI 10.17504/protocols.io.2srged6. 10X Chromium single-cell 3’ v3: manufacturer’s protocol version #CG000183_ChromiumSingleCell3′_v3_UG_Rev-A. |  |
|  |  |  |
| **Experimental study design (statistics details) *** | | |
| **For in vivo studies: State whether and how the following have been done** | **Indicate where provided: section/figure legend. If it could have been done, but was not, write “not done”** | **N/A** |
| Sample size determination | Not done. The sample size of 11 marmosets reflect the availability of marmosets that could be used in the study (part of the marmoset brain atlas project or neonates that died after birth). |  |
| Randomisation |  | N/A |
| Blinding |  | N/A |
| Inclusion/exclusion criteria | Not done. All adult marmosets had no known previous disease and were selected as part of the marmoset brain atlas project. The three neonates were included despite unknown cause of death (not excluded). |  |
|  |  |  |
| **Sample definition and in-laboratory replication** | **Indicate where provided: section/figure legend** | **N/A** |
| State number of times the experiment was replicated in the laboratory. | Results/Methods/Table 1: chimerism was independently assessed in 11 marmosets (137 brain tissue samples, plus blood, liver and kidney), each an independent biological replicate; consistent myeloid-lineage-specific chimerism was observed across all animals. |  |
| Define whether data describe technical or biological replicates. | Data represent biological replicates (independent animals and independent tissue dissections/libraries per animal; Table 1); no technical (repeat-library) replicates were generated for a given sample. |  |
|  |  |  |
| **Ethics** | **Indicate where provided: section/submission form** | **N/A** |
| Studies involving human participants: State details of authority granting ethics approval (IRB or equivalent committee(s), provide reference number for approval. |  | N/A |
| Studies involving experimental animals: State details of authority granting ethics approval (IRB or equivalent committee(s), provide reference number for approval. | Methods (Ethical compliance): approved by the Massachusetts Institute of Technology IACUC, protocol number 051705020. |  |
| Studies involving specimen and field samples: State if relevant permits obtained, provide details of authority approving study; if none were required, explain why. |  | N/A |
|  |  |  |
| **Dual Use Research of Concern (DURC)** | **Indicate where provided: section/submission form** | **N/A** |
| If study is subject to dual use research of concern regulations, state the authority granting approval and reference number for the regulatory approval. |  | N/A |

**Analysis:**

| **Attrition** | **Indicate where provided: section/figure legend** | **N/A** |
| --- | --- | --- |
| Describe whether exclusion criteria were pre-established. Report if sample or data points were omitted from analysis. If yes, report if this was due to attrition or intentional exclusion and provide justification. | Methods (Donor-of-origin analysis and detection of host-sibling doublets; Additional filtering for microglia and macrophage clusters): pre-established filtering criteria (minimum UMI, doublet detection/removal, sub-cluster filtering of non-chimeric, likely misclassified cells) are described, with excluded nuclei and rationale reported (Appendix 1-table 4, Fig. 2-figure supplement 4, Fig.2-figure supplement 5). |  |
|  |  |  |
| **Statistics** | **Indicate where provided: section/figure legend** | **N/A** |
| Describe statistical tests used and justify choice of tests. | Methods (Gene expression analysis of host and sibling meta cells; Binomial generalized linear mixed-effects model analysis; Latent factor analysis): differential expression tested with the edgeR binomTest, chimerism-vs-covariate relationships tested with binomial generalized linear mixed-effects models (glmer), and PEER latent-factor linear regression; rationale for each test given in the text. |  |
|  |  |  |
| **Data availability** | **Indicate where provided: section/submission form** | **N/A** |
| For newly created and reused datasets, the manuscript includes a data availability statement that provides details for access (or notes restrictions on access). | Methods (Data availability): a data availability statement is provided describing access to all newly generated sequencing datasets. |  |
| When newly created datasets are publicly available, provide accession number in repository OR DOI and licensing details where available. | Methods (Data availability): snRNA-seq datasets deposited at the BICCN NeMO Archive (https://assets.nemoarchive.org/dat); whole-genome sequencing deposited at NIH SRA, BioProject PRJNA1068102. |  |
| If reused data is publicly available provide accession number in repository OR DOI, OR URL, OR citation. | Methods (Data availability): publicly available marmoset single-cell data from https://assets.nemoarchive.org/dat-1je0mn3 |  |
|  |  |  |
| **Code availability** | **Indicate where provided: section/figure legend** | **N/A** |
| For any computer code/software/mathematical algorithms essential for replicating the main findings of the study, whether newly generated or re-used, the manuscript includes a data availability statement that provides details for access or notes restrictions. | Methods (Software availability): a statement is provided describing all software used, all of which is publicly available. |  |
| Where newly generated code is publicly available, provide accession number in repository, OR DOI OR URL and licensing details where available. State any restrictions on code availability or accessibility. |  | N/A |
| If reused code is publicly available provide accession number in repository OR DOI OR URL, OR citation. | Methods (Software availability): bwa (https://github.com/lh3/bwa), GATK (https://gatk.broadinstitute.org), BCFtools (https://github.com/samtools/bcftools), samtools (http://www.htslib.org/download), Picard Tools, Seurat, scType, SCTransform and PEER, with URLs provided for each. The Drop-seq software suite, including the Dropulation and Census-seq tools used for donor-of-origin/chimerism analysis, is publicly available at https://github.com/broadinstitute/Drop-seq. |  |

**Reporting:**

The MDAR framework recommends adoption of discipline-specific guidelines, established and endorsed through community initiatives.

| **Adherence to community standards** | **Indicate where provided: section/figure legend** | **N/A** |
| --- | --- | --- |
| State if relevant guidelines (e.g., ICMJE, MIBBI, ARRIVE, STRANGE) have been followed, and whether a checklist (e.g., CONSORT, PRISMA, ARRIVE) is provided with the manuscript. |  | N/A |

* We provide the following guidance regarding transparent reporting and statistics; we also refer authors to [Ten common statistical mistakes to watch out for when writing or reviewing a manuscript](https://doi.org/10.7554/eLife.48175).

**Sample-size estimation**

- You should state whether an appropriate sample size was computed when the study was being designed
- You should state the statistical method of sample size computation and any required assumptions
- If no explicit power analysis was used, you should describe how you decided what sample (replicate) size (number) to use

**Replicates**

- You should report how often each experiment was performed
- You should include a definition of biological versus technical replication
- The data obtained should be provided and sufficient information should be provided to indicate the number of independent biological and/or technical replicates
- If you encountered any outliers, you should describe how these were handled
- Criteria for exclusion/inclusion of data should be clearly stated
- High-throughput sequence data should be uploaded before submission, with a private link for reviewers provided (these are available from both GEO and ArrayExpress)

**Statistical reporting**

- Statistical analysis methods should be described and justified
- Raw data should be presented in figures whenever informative to do so (typically when N per group is less than 10)
- For each experiment, you should identify the statistical tests used, exact values of N, definitions of center, methods of multiple test correction, and dispersion and precision measures (e.g., mean, median, SD, SEM, confidence intervals; and, for the major substantive results, a measure of effect size (e.g., Pearson's r, Cohen's d)
- Report exact p-values wherever possible alongside the summary statistics and 95% confidence intervals. These should be reported for all key questions and not only when the p-value is less than 0.05.

**Group allocation**

- Indicate how samples were allocated into experimental groups (in the case of clinical studies, please specify allocation to treatment method); if randomization was used, please also state if restricted randomization was applied
- Indicate if masking was used during group allocation, data collection and/or data analysis
