# Supplementary material for: Sibling chimerism among microglia in marmosets
Source: eLife. 2026 Aug 14;13:RP93640. doi: 10.7554/eLife.93640 (PMC13476068; doi:10.7554/eLife.93640)
Supplement: Peer review history

# Editor report: eLife Assessment

**DOI:** 10.7554/eLife.93640.3.sa0

**Authors:** Vincent J Lynch

**Affiliations:** University at Buffalo, State University of New York United States

**Keywords:** Compelling, Fundamental

This **fundamental** study substantially advances our understanding of sibling chimerism in marmosets by demonstrating that chimerism is limited to hematopoietic cells. The evidence supporting these findings is **compelling**, demonstrated through comprehensive analyses, including single-cell RNA-seq data from multiple individuals and tissues. A few minor concerns were successfully addressed in a revision. The work will be of broad interest to many fields of biology.

# Referee report: Reviewer #1 (Public review)

**DOI:** 10.7554/eLife.93640.3.sa1

Summary:

Del Rosario et al characterized the extent and cell types of sibling chimerism in marmosets. To do so, they took advantage of the thousands of SNPs that are transcribed in single-nucleus RNA-seq (snRNA-seq) data to identify the sibling genotype of origin for all sequenced cells across 4 tissues (blood, liver, kidney, and brain) from many marmosets. They found that chimerism is prevalent and widespread across tissues in marmosets, which has previously been shown. However, their snRNA-seq approach allowed them to identify precisely which cells were of sibling origin, and which were not. In doing so they definitively show that sibling chimerism across tissues is limited to cells of myeloid and lymphoid lineages. The authors then focus on a large sample of microglia sequenced across many brain regions to quantify: (1) variation in chimerism across brain regions in the same individual, and (2) the relative importance of genetic vs. environmental context on microglia function/identity. (1) Much like across different tissues in the same individual, they found that the proportion of chimeric microglia varies across brain regions collected from the same individuals (as well as differing from the proportion of sibling cells found in blood of the same animals), suggesting that cells from different genetic backgrounds may differ in their recruitment and/or proliferation across regions and local tissue contexts, or that this may be linked to stochastic bottleneck effects during brain development. (2) Their (admittedly smaller sample size) analyses of host-sibling gene expression showed that the local environment dominates genotype. All told, this thoughtful and thorough manuscript accomplishes two important goals. First, it all but closes a previously open question on the extent and cell origins of sibling chimerism. Second, it sets the stage for using this unique model system to examine, in a natural context, how genetic variation in microglia may impact brain development, function, and disease.

The conclusions of this paper are well supported by the data, and the authors exert appropriate care when extrapolating their results that come from smaller samples. However, there are a few concerns that should be addressed.

The "modest correlation" mentioned in lines 170-172 does not take into account the uncertainty in estimates of each chimeric cell proportion (although the plot shows those estimates nicely). This is particularly important for the macrophages, which are far less abundant. Perhaps a more appropriate way to model this would be in a binomial framework (with a random effect for individual of origin). Here, you could model sibling identity of each macrophage as a function of the proportion of sibling-origin microglia and then directly estimate the percent variance explained.

A similar (albeit more complicated because of the number of regions being compared) approach could be applied to more rigorously quantify the variation in chimerism across brain regions (L198-215; Fig 4). This would also help to answer the question of whether specific brain regions are more "amenable" to microglia chimerism than others.

While the sample size is small, it would be exciting to see if any microglia eQTL are driven by sibling chimerism across the marmosets.

L290-292: The authors should propose ways in which they could test the two different explanations proposed in this paragraph. For instance, a simulation-based modeling approach could potentially differential more stochastic bottleneck effects from recruitment-like effects.

While intriguing, the gene expression comparison (Fig 5) is extremely underpowered. It would be helpful to clarify this and note the statistical thresholds used for identifying DEGs (the black points in the figure).

Comments on revisions:

The authors have thoroughly addressed all my suggestions.

# Referee report: Reviewer #2 (Public review)

**DOI:** 10.7554/eLife.93640.3.sa2

Summary:

This manuscript reports a novel and quite important study of chimerism among common marmosets. As the authors discuss, it has been known for years that marmosets display chimerism across a number of tissues. However, as the authors also recognize, the scope and details of this chimerism have been controversial. Some prior publications have suggested that the chimerism only involves cells derived from hematopoietic stem cells, while other publications have suggested more cell types can also be chimeric, including a wide range of cell types present in multiple organs. The present authors address this question and several other important issues by using snRNA-seq to track the expression of host and sibling-derived mRNAs across multiple tissues and cell types. The results are clear and provide convincing evidence that for the various organs analyzed, all chimeric cells are derived from hematopoietic cell lineages.

This work will have impact on studies using marmosets to investigate various biological questions, but will have biggest impact on neuroscience and studies of cellular function within the brain. The demonstration that microglia and macrophages from different siblings from a single pregnancy, with different genomes expressing different transcriptomes, are commonly present within specific brain structures of a single individual opens a number of new opportunities to study microglia and macrophage function as well as interations between microglia, macrophages and other cell types.

Strengths:

The paper has a number of important strengths. This analysis employs the first unambiguous approach providing a clear answer to the question of whether sibling-derived chimeric cells arise only from hematopoietic lineages or from a wider array of embryonic sources. That is a long-standing open question and these snRNA-seq data seem to provide a clear answer, at least for brain and liver and kidney. In addition, the present authors investigate quantitative variation in chimeric cell proportions across several dimensions, comparing the proportion of chimeric cells across individual marmosets, across organs within an individual and across brain regions within an individual. All these are significant questions, and the answers have important implications for multiple research areas. Marmosets are increasingly being used for a range of neuroscience studies, and a better understanding of the process that leads to chimerism of microglia and macrophages in the marmoset brain is a valuable and timely contribution. But this work also has implications for other lines of study such as defining embryological and development processes and the potential to track specific cell populations within genetically engineered marmosets. Third, the snRNA-seq data will be made available through Brain Initiative NeMO portal and the software used to quantify host vs. sibling cell proportions in different biosamples will be available through Github.

Comments on revisions:

Several minor weaknesses have been addressed by the authors in a revision of the original manuscript. Each of my concerns and perceived weaknesses regarding the initial submission have been satisfactorily addressed in the revision.

# Author comment: Author response

**DOI:** 10.7554/eLife.93640.3.sa3

**Authors:** Ricardo CH del Rosario, Fenna M Krienen, Qiangge Zhang, Melissa Goldman, Curtis Mello, Alyssa Lutservitz, Kiku Ichihara, Alec Wysoker, James Nemesh, Guoping Feng, Steven A McCarroll

**Affiliations:** Broad Institute Cambridge United States; Princeton University Princeton United States; Massachusetts Institute of Technology Cambridge United States; Broad Institute Cambridge United States; Harvard Medical School Cambridge United States; Harvard Medical School Boston United States; Broad Institute Cambridge United States; Broad Institute Cambridge United States; Broad Institute Cambridge United States; Massachusetts Institute of Technology Cambridge United States; Harvard Medical School Boston United States

The following is the authors’ response to the original reviews.

**Public Reviews:**

**Reviewer #1 (Public review):**

Summary:

Del Rosario et al characterized the extent and cell types of sibling chimerism in marmosets. To do so, they took advantage of the thousands of SNPs that are transcribed in single-nucleus RNA-seq (snRNA-seq) data to identify the sibling genotype of origin for all sequenced cells across 4 tissues (blood, liver, kidney, and brain) from many marmosets. They found that chimerism is prevalent and widespread across tissues in marmosets, which has previously been shown. However, their snRNA-seq approach allowed them to identify precisely which cells were of sibling origin, and which were not. In doing so they definitively show that sibling chimerism across tissues is limited to cells of myeloid and lymphoid lineages. The authors then focus on a large sample of microglia sequenced across many brain regions to quantify: (1) variation in chimerism across brain regions in the same individual, and (2) the relative importance of genetic vs. environmental context on microglia function/identity.

(1) Much like across different tissues in the same individual, they found that the proportion of chimeric microglia varies across brain regions collected from the same individuals (as well as differing from the proportion of sibling cells found in the blood of the same animals), suggesting that cells from different genetic backgrounds may differ in their recruitment and/or proliferation across regions and local tissue contexts, or that this may be linked to stochastic bottleneck effects during brain development.

(2) Their (admittedly smaller sample size) analyses of host-sibling gene expression showed that the local environment dominates genotype.

All told, this thoughtful and thorough manuscript accomplishes two important goals. First, it all but closes a previously open question on the extent and cell origins of sibling chimerism. Second, it sets the stage for using this unique model system to examine, in a natural context, how genetic variation in microglia may impact brain development, function, and disease.

The conclusions of this paper are well supported by the data, and the authors exert appropriate care when extrapolating their results that come from smaller samples. However, there are a few concerns that should be addressed.

The "modest correlation" mentioned in lines 170-172 does not take into account the uncertainty in estimates of each chimeric cell proportion (although the plot shows those estimates nicely). This is particularly important for the macrophages, which are far less abundant. Perhaps a more appropriate way to model this would be in a binomial framework (with a random effect for individuals of origin). Here, you could model the sibling identity of each macrophage as a function of the proportion of sibling-origin microglia and then directly estimate the percent variance explained.

We appreciate this good suggestion. We performed an analysis along these lines, and found that it supported the conclusion of a lack of strong relationship between microglial and macrophage chimerism. In particular (and as we now have added to the Methods):

“To perform an analysis of Fig. 2D that takes into account the uncertainty in the estimate of the chimeric cell proportion, we performed a binomial generalized linear mixed-effects model analysis in R using the command glmer (y~(1|indiv) + chimerism_micro, family=binomial), where y is a vector (of length 1,333) containing the genomic identity of each macrophage (either host or twin), 1|indiv models a random effect for the identity of each animal, and chimerism_micro is the microglia chimerism of the animal’s brain. The fixed effects probability of chimerism_micro was 0.795, indicating that microglial chimerism fraction was not statistically significant as a predictor for macrophage chimerism fraction. The estimate for the intercept was -0.8115 and the estimate for chimerism_micro was 0.3106, which indicates that the probability of a cell is a macrophage given the microglia chimerism fraction was only 0.57 (plogis (-0.8115+0.3106)).”

We have added the following in the main text:

“We investigated further by performing a statistical test that takes into account the uncertainty in the estimates of the chimeric cell proportion using a binomial framework (Methods); in this analysis, microglia chimerism fraction was not a statistically significant predictor of macrophage chimerism fraction (Methods). This suggests that in addition to the cell’s genome, other factors such as local host environment play a role in differential recruitment, proliferation or survival of the sibling cells. (We note that macrophages often transit the fluid-filled perivascular space, with a substantially different migration history and arrival dynamics than microglia.)”

Given this new analysis, and our original observation that the Pearson correlation was only 0.31, we believe that other factors in addition to the cell’s genome play a role in differential recruitment or survival of sibling cells.

A similar (albeit more complicated because of the number of regions being compared) approach could be applied to more rigorously quantify the variation in chimerism across brain regions (L198-215; Figure 4). This would also help to answer the question of whether specific brain regions are more "amenable" to microglia chimerism than others.

We performed the analysis along these lines and added the following in the Methods section:

“We used the same framework to further analyze Fig. 4. We included brain region as a covariate in the binomial framework: glmer (y~(1|indiv) + brain_reg + assay, family=binomial), where, y is a vector (of length 48,439) containing the genomic identity of each microglia, and assay is either “Drop-seq” or “10X”. The brain regions assayed in Fig. 4 are the cortex, hippocampus, hypothalamus, striatum, thalamus, and basal forebrain. All these brain regions were statistically significant predictors for microglia chimerism fraction (all *P*-values<2x10^-16^), supporting the conclusion that chimerism varies across brain regions. We also re-analyzed Supplementary Fig. 4 (Fig. 4B in original manuscript) using the same framework and found that 18 out of 27 brain substructures were statistically significant predictors for microglia chimerism fraction.”

We have added the following sentences in the main text:

“We used the binomial generalized linear mixed-model framework and found that all brain regions were statistically significant predictors for microglia chimerism fraction, supporting the conclusion that chimerism varies across brain regions (Methods).

Analysis of finer brain substructures showed a similar result (Supplementary Fig. 4; the binomial generalized linear mixed-model framework determined that 18 out of 27 brain substructures were statistically significant as predictors for microglia chimerism fraction, Methods).”

While the sample size is small, it would be exciting to see if any microglia eQTL are driven by sibling chimerism across the marmosets.

We like this idea, but our study is underpowered for eQTL analysis since we only have 14 data points in the correlation analysis (eight cases in which an animal’s brain hosted microglia derived from a single sibling, plus three cases in which an animal’s brain hosted microglia derived from two siblings, collectively allowing 8 + (2*3)=14 pairwise analyses).

L290-292: The authors should propose ways in which they could test the two different explanations proposed in this paragraph. For instance, a simulation-based modeling approach could potentially differentiate more stochastic bottleneck effects from recruitment-like effects.

While intriguing, the gene expression comparison (Figure 5) is extremely underpowered. It would be helpful to clarify this and note the statistical thresholds used for identifying DEGs (the black points in the figure).

We agree; to help clarify this for readers, we added the following sentence at the end of the paragraph discussing Fig. 5A-C.

“In all eleven individual marmosets, analysis identified genes whose differential expression distinguished microglia with the two sibling genomes (hundreds of genes in total), documenting a substantial effect of sibling genetic differences on microglial gene expression. However, we did not find any gene whose expression level recurrently distinguished “host” microglia (microglia with the same genome as neural cell types) from “guest” microglia (microglia with the sibling genome), aside from the XIST gene (a proxy for sibling sex differences, which were of course common) (Supplementary Fig. 5, Fig. 5A-C). In other words, although there were always gene-expression differences between sibling microglia, none of them consistently distinguished between host and guest microglia, suggesting that they were instead due to sibling genetic differences. We note that both analyses are power-limited, as the number of microglia in most animals, especially guest microglia, were modest (Supplementary Fig. 5); thus, we cannot rule out the possibility that there may be one or more genes whose expression levels reflect developmental histories (host vs. guest origin), just as there are likely far more genes (than the hundreds we identified) that can have sibling expression differences due e.g. to genetic differences between siblings. We sought to increase power (beyond single-gene analysis) by using latent factor analysis (Ling et al., 2024) to identify and quantify the expression of microglial gene-expression programs; however, even this analysis did not find any gene expression programs that exhibited consistent host-twin differences in expression levels (Methods).”

And in the caption of Fig. 5A-C, we have included the statistical threshold for identifying DEGs:

“In (A) to (C), each point represents a gene; its location on the plot represents the level of expression of that gene among microglia with two different genomes in the same animal. x- and y-axes: normalized gene expression levels (number of transcripts per 100,000 transcripts). FC: fold-change of gene expression, female/male for XIST. Fold-change and *P*-values were calculated using the binomTest method from the edgeR package (Robinson et al., 2010). Differentially expressed genes (black dots) were defined as: FDR *Q*-value<0.05 and fold-change>1.5 (in either direction) and the gene must be expressed in at least 10% of at least one of the two sets of microglia being compared.”

**Reviewer #2 (Public review):**

Summary:

This manuscript reports a novel and quite important study of chimerism among common marmosets. As the authors discuss, it has been known for years that marmosets display chimerism across a number of tissues. However, as the authors also recognize, the scope and details of this chimerism have been controversial. Some prior publications have suggested that the chimerism only involves cells derived from hematopoietic stem cells, while other publications have suggested more cell types can also be chimeric, including a wide range of cell types present in multiple organs. The present authors address this question and several other important issues by using snRNA-seq to track the expression of host and sibling-derived mRNAs across multiple tissues and cell types. The results are clear and provide strong evidence that all chimeric cells are derived from hematopoietic cell lineages.

This work will have an impact on studies using marmosets to investigate various biological questions but will have the biggest impact on neuroscience and studies of cellular function within the brain. The demonstration that microglia and macrophages from different siblings from a single pregnancy, with different genomes expressing different transcriptomes, are commonly present within specific brain structures of a single individual opens a number of new opportunities to study microglia and macrophage function as well as interactions between microglia, macrophages, and other cell types.

Strengths:

The paper has a number of important strengths. This analysis employs the first unambiguous approach providing a clear answer to the question of whether sibling-derived chimeric cells arise only from hematopoietic lineages or from a wider array of embryonic sources. That is a long-standing open question and these snRNA-seq data seem to provide a clear answer, at least for the brain, liver, and kidney. In addition, the present authors investigate quantitative variation in chimeric cell proportions across several dimensions, comparing the proportion of chimeric cells across individual marmosets, across organs within an individual, and across brain regions within an individual. All these are significant questions, and the answers have important implications for multiple research areas. Marmosets are increasingly being used for a range of neuroscience studies, and a better understanding of the process that leads to the chimerism of microglia and macrophages in the marmoset brain is a valuable and timely contribution. But this work also has implications for other lines of study. Third, the snRNA-seq data will be made available through the Brain Initiative NeMO portal and the software used to quantify host vs. sibling cell proportions in different biosamples will be available through GitHub.

Weaknesses:

I find no major weaknesses, but several minor ones. First, the main text of the manuscript provides no information about the specific animals used in this study, other than sex. Some basic information about the sources of animals and their ages at the time of study would be useful within the main paper, even though more information will be available in the supplementary material.

We moved the table containing animal information (age at time of study, sex, source, tissues analyzed) from Supplementary Table 1 into the main text as Table 1. We also added the following sentences starting on line 140:

“Brain snRNA-seq was performed on 11 animals (6 adults, 3 neonates and 1 six months old; Table 1). All were unrelated except for CJ006 and CJ007 which are birth siblings, and CJ025 and CJ026 which are (non-birth) siblings. All animals come from the three main marmoset colonies that comprise the animals in our facilities: New England Primate Research Center (NEPRC), CLEA Japan, and from a non-clinical contract research organization in Massachusetts. All adult marmosets had no known previous disease and were selected as part of a larger project to create a single cell atlas of the marmoset brain. The three neonates had died shortly after birth due to unknown reasons and were subsequently selected for snRNA-seq analysis.”

Second, it is not clear why only 14 pairs of animals were used for estimating the correlation of chimerism levels in microglia and macrophages. Is this lower than the total number of pairwise comparisons possible in order to avoid using non-independent samples? Some explanation would be helpful.

Only birth siblings (twins and triplets) can be meaningfully included in this analysis. The 14 pairs of animals we used to estimate the correlation of chimerism levels in microglia and macrophages included all pairs that we could use for this analysis: eight cases in which an animal’s brain hosted microglia derived from a single sibling, plus three cases in which an animal’s brain hosted microglia derived from two siblings, collectively allowing 8 + (2*3)=14 pairwise analyses.

Finally, I think more analysis of the consistency and variability of gene expression in microglia across different regions of the brain would be valuable. Are there genetic pathways expressed similarly in host and sibling microglia, regardless of region of the brain? Are there pathways that are consistently expressed differently in host vs sibling microglia regardless of brain region?

For brain-region differences in microglial gene expression, we are under-powered and would only be scratching the surface of a question (interesting but beyond the focus and scope of this paper) that needs deeper experimental sampling.

For the questions about sibling-sibling differences (regardless of which sibling is host) and recurring host-sibling differences, we can do a stronger analysis, because these analyses have similar power to each other. We describe this analysis in the revised manuscript as follows:

“In all eleven individual marmosets, analysis identified genes whose differential expression distinguished microglia with the two sibling genomes (hundreds of genes in total), documenting a substantial effect of sibling genetic differences on microglial gene expression. However, we did not find any gene whose expression level recurrently distinguished “host” microglia (microglia with the same genome as neural cell types) from “guest” microglia (microglia with the sibling genome), aside from the XIST gene (a proxy for sibling sex differences, which were of course common) (Supplementary Fig. 5, Fig. 5A-C). In other words, although there were always gene-expression differences between sibling microglia, none of them consistently distinguished between host and guest microglia, suggesting that they were instead due to sibling genetic differences. We note that both analyses are power-limited, as the number of microglia in most animals, especially guest microglia, were modest (Supplementary Fig. 5); thus, we cannot rule out the possibility that there may be one or more genes whose expression levels reflect developmental histories (host vs. guest origin), just as there are likely far more genes (than the hundreds we identified) that can have sibling expression differences due e.g. to genetic differences between siblings.”

We also, as suggested, tried to get beyond single-gene analyses to expression of programs/pathways, by performing latent factor analysis on the single-cell gene expression measurements.

“Following the method described in (Ling et al., 2024), we performed latent factor analysis using the probabilistic estimation of expression residuals (PEER, Stegle et al., 2010) on the gene-by-donor matrix expression of microglia. We started by creating a gene-by-cell matrix of microglia gene expression from all animals, and we normalized the matrix using SCT transform version 2 (Choudhary and Satija, 2022) with 3000 variable features. We obtained the Pearson residuals from SCT normalization and summed up the residuals across cells with the same genome to obtain a gene-by-donor matrix of expression measurements of microglia. We used this matrix as input to PEER and ran the tool with a provided number of factors from 9 to 12. For each gene-expression latent factor, to evaluate whether host/sibling identity had a consistent effect on expression levels, we performed a linear regression with host/sibling identity using glm(peer_factor_k ~ host_or_twin). For all factors, the *P*-values for the effect of host_or_twin were all insignificant (greater than 0.1), indicating that no PEER factor associated with host-vs-twin identity. Thus, our results found no large-scale gene expression program that was consistently expressed differently between hosts and twins.”

We have added the text above to the Methods section, and we added the following at the end of the section on Gene-expression comparisons of host- to sibling-derived microglia (lines 264-267):

“We sought to increase power (beyond single-gene analysis) by using latent factor analysis (Ling et al., 2024) to identify and quantify the expression of microglial gene-expression programs; however, even this analysis did not find any gene expression programs that exhibited consistent host-twin differences in expression levels (Methods).”

Gene-expression pathways/factors did (within some animals) did show host-twin differences in expression levels, but without a consistent host-twin direction of effect that was shared across the many host-twin comparisons. In particular, we used the PEER analysis that we have performed above and calculated the host-sibling expression level difference for each latent factor. Many factors differed in expression in individual cases, though none did so in all cases nor in a consistent-sign manner:

**Author response image 1.** Difference between host and sibling expression of gene-expression latent factors for each of the 12 factors computed (using PEER) from the single-cell dataset. For a given factor, the factor expression value of the sibling-genome cells is subtracted from that of the host-genome cells and the difference is divided by the maximum of the absolute value of all elements in that factor.

**Recommendations for the authors:**

**Reviewer #1 (Recommendations for the authors):**

In the introduction (line 62), the authors mention that chimerism might have shaped behavior in marmosets (and perhaps been selected for). It would be helpful to see this revisited in the discussion. Is it possible that additional genetic variation in immune cells (resident and circulating) provides adaptive benefits and/or disease resistance? In the case of microglia, could the proportion of sibling cells be related (either positively or negatively) to local/regional pathology?

We liked this suggestion and have added the following in the Discussion:

“Chimerism could also enable interesting future analyses of whether there are adaptive benefits of chimerism in marmoset immune cells, among whom chimerism could in principle allow presentation of a wider variety of antigens for adaptive immunity. In a recent outbreak of yellow fever in Brazil in 2016-2018, marmosets were found to be less susceptible than other primates that lack immune system chimerism, including the howler monkeys (*Alouatta*), robust capuchins (*Sapajus*), and titi monkeys (*Callicebus*) (de Azebedo Fernandes, et al., 2021). In studying future outbreaks in marmosets, one could use single-cell RNA-seq and the methods described here to study how genetically distinct immune cells (in the same animal) have differentially migrated to affected tissues and/or assumed "activated" immune cell states. Recent innovations in spatial transcriptomics with sequencing readouts (that detect SNP alleles) may also make it possible to identify any differential recruitment of genetically distinct immune cells to focal infection sites.”

Minor comments:

L300 delete "temporal.”

We have revised the text accordingly.

L305: "more-restricted" should not be hyphenated.

We have revised the text accordingly.

L309: "from the non-cell" - delete "the.”

We have revised the text accordingly.

L367: Louvain, not Louvaine.

We have revised the text accordingly.

Figure 2B can be removed - it does not add much information and takes up a lot of space.

We have moved Figure 2B to panel J Supplementary Fig. 1 (it is now displayed together with all other animals).

The same can be said for Figure 4B, which is too tiny. There might be more effective ways to show this variation across animals.

We have moved Figure 4B to Supplementary Fig. 4 and we have increased the font sizes to make the text in the figures more readable.

**Reviewer #2 (Recommendations for the authors):**

I would suggest providing some basic information about the sources of study animals within the main text. At a minimum, it would be useful to state which colonies are represented in the data, and if there is anything significant about the individual animal histories (e.g. prior exposure to surgical intervention or infectious disease). I believe this basic information should be in the main text, despite the inclusion of a broader range of information in the supplements.

We appreciate this suggestion and revised lines 143 to 149 of the main text as follows:

“All animals come from the three main marmoset colonies that comprise the animals in our facilities: New England Primate Research Center (NEPRC), CLEA Japan, and from a non-clinical contract research organization. All adult marmosets had no known previous disease and were selected as part of a larger project to create a single-cell atlas of the marmoset brain (Krienen et al., 2020; Krienen et al., 2023). The three neonates died shortly after birth due to unknown reasons and were subsequently selected for snRNA-seq analysis.”

I would include the species name (Callithrix jacchus) in line 48.

“On lines 47-48, we now indicate the name of the genus: “Chimerism is common, however, in the *Callitrichidae* family that consists of the marmosets (*Callithrix*) and their close relatives the tamarins (*Saguinus*)...”

Then on line 65, we now indicate the species name: “Here, we analyze chimerism in the common marmoset (*Callithrix jacchus*) brain, liver, kidney and blood,...”

The word "organisms" in line 59 should be "organs.”

We have modified the text accordingly.

Lines 100-101: I would suggest this would be clearer to readers if it read: "The relative likelihoods of the original source of each cell could be strongly...".

We have modified the text accordingly.
